# Endogenous estrogen receptor modulating oxysterols and breast cancer prognosis: Results from the MARIE patient cohort

**DOI:** 10.1038/s41416-023-02315-w

**Published:** 2023-06-24

**Authors:** Nina Sophia Decker, Theron Johnson, Sabine Behrens, Nadia Obi, Rudolf Kaaks, Jenny Chang-Claude, Renée Turzanski Fortner

**Affiliations:** 1 Division of Cancer Epidemiology, German Cancer Research Center (DKFZ), Im Neuenheimer Feld 280, 69120 Heidelberg, Germany; 2 Medical Faculty Heidelberg, Heidelberg University, Heidelberg, Germany; 3 Institute for Medical Biometry and Epidemiology, University Medical Center Hamburg-Eppendorf, Martinistrasse 52, 20246 Hamburg, Germany; 4 University Cancer Center Hamburg, University Medical Center Hamburg-Eppendorf, Martinistrasse 52, 20246 Hamburg, Germany; 5 Department of Research, Cancer Registry of Norway, Ullernchausseen 64, 0379 Oslo, Norway

**Keywords:** Prognostic markers, Breast cancer, Breast cancer

## Abstract

**Background:**

27-hydroxycholesterol (HC) and 25-HC were identified as endogenous selective estrogen receptor modulators (SERMs) and estrogen receptor (ER) modulators, respectively. They are hypothesized to play a role in multiple physiologic processes and pathologies, including breast cancer development and progression.

**Methods:**

We evaluated circulating 27-HC and 25-HC, and outcomes following a breast cancer diagnosis in 2282 women from the MARIE study over median follow-up of 11.6 years. 27-HC and 25-HC were quantified by liquid chromatography–mass spectrometry. We calculated hazard ratios (HR) and 95% confidence intervals [CI] using multivariable Cox Proportional Hazards regression.

**Results:**

We observed no associations between 27-HC and breast cancer prognosis overall. Associations between 27-HC and survival differed by circulating estradiol concentrations and endocrine therapy, but not by hormone receptor status. Among women with estradiol levels below the median (0.08 nM), 27-HC was associated with higher risk of all-cause mortality (HR_log2_ = 1.80 [1.20–2.71]) and breast cancer-specific mortality (HR_log2_ = 1.95 [1.14-3.31]). No associations were observed in women with estradiol levels above the median. Higher 25-HC levels were associated with lower risk of recurrence (HR_log2_ = 0.87 [0.77-0.98]).

**Conclusion:**

Associations between 27-HC and breast cancer prognosis varied by circulating estradiol levels and endocrine therapy. Less consistent results were observed for 25-HC.

## Background

Breast cancer is the most commonly diagnosed cancer in women and the leading cause of cancer death in women worldwide [1]. Despite the overall favorable survival and available targeted treatments, in particular for estrogen receptor (ER)-positive or HER2-positive breast cancers, resistance to treatment is well recognized and novel potentially targetable pathways are needed [2]. Emerging data suggests that oxysterols, oxidized derivates of cholesterol, including 27-hydroxycholesterol (HC) and 25-HC, play a role in various chronic conditions including cardiovascular diseases [3, 4], neurological diseases [3, 4], and cancer [4, 5]. 27-HC and 25-HC were identified as endogenous estrogen receptor (ER) modulators, with 27-HC named as the first endogenous selective ER modulator (SERM) [6]. 27-HC has been of particular interest in breast cancer both in experimental models [7–9] and in clinical and epidemiological studies [8–14].

Previous research suggests that higher concentrations of circulating pre-diagnosis 27-HC are associated with lower risk of postmenopausal breast cancer [10]. To our knowledge, only one study has directly addressed circulating 27-HC, 25-HC, and breast cancer survival in a small patient cohort (*n* = 58) [14], while several other studies indirectly assessed the association between 27-HC and prognosis by evaluating the levels of protein or mRNA expression of the enzymes converting cholesterol to 27-HC (CYP27A1) or metabolizing 27-HC to downstream metabolites (CYP7B1) [8, 9, 11–13]. Results of these studies were conflicting with three studies reporting poorer prognosis with higher CYP27A1 or lower CYP7B1 expression [8, 9, 13], and two studies reporting better prognosis with higher CYP27A1 expression [11, 12].

Following the experimental evidence on oxysterols in breast cancer and the inconsistent findings in breast cancer patients, we aimed to investigate the association between circulating 27-HC, 25-HC, and breast cancer prognosis in a large breast cancer cohort to contribute to better understanding the link between cholesterol metabolism and breast cancer survival following a breast cancer diagnosis. Due to hypothesized differences in associations by estradiol concentrations and endocrine therapy, we investigated the associations between 27-HC, 25-HC, and breast cancer prognosis by circulating estradiol levels and exogenous SERM and aromatase inhibitor (AI) use.

## Methods

**Fig. 1:**
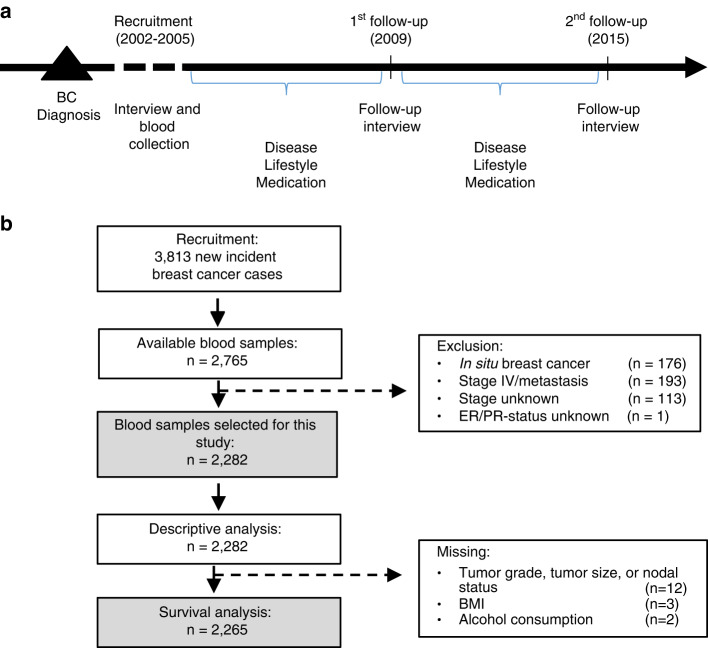
Study design and data collection. **a** Study design of the MARIE breast cancer patient cohort: Participants were recruited post-diagnosis between 2002 and 2005, and provided a blood sample and information on disease and lifestyle at baseline; follow-up (FU) information on disease, lifestyle, medication were retrieved in 2009 (FU1) and 2015 (FU2). **b** Flow-chart of inclusion and exclusion criteria for participants of the MARIE breast cancer patient cohort study for analysis related to 27-hydroxcholesterol, 25-hydroxycholesterol, and breast cancer prognosis.

We conducted this study in the Mammary Carcinoma Risk factor Investigation (MARIE), a large population-based patient cohort. Details of the study have been published previously [15]. In brief, 3813 breast cancer patients aged 50–74 years at baseline with a histologically confirmed primary invasive (stages I-IV) or in situ carcinoma diagnosis prior to recruitment were enrolled between August 2002 and September 2005 in two regions in Germany, the city of Hamburg and the Rhein–Neckar–Karlsruhe (RNK) region (Fig. 1a). A total of 2771 participants provided a blood sample at baseline, of which 2765 blood samples were available for this study. Patient and lifestyle characteristics were obtained via baseline interviews and follow-up questionnaires. For this study, women with in situ breast cancers, metastasis at diagnosis (stage IV disease), previous tumors other than breast cancer, or missing hormone receptor status were excluded. In total, 2282 participants of the MARIE study with available blood sample were included in the present study. Selection of the study sample is shown in (Fig. 1b).

### Endocrine therapy use

Due to the hypothesized role of 27-HC as an endogenous SERM, we evaluated associations of oxysterols with breast cancer prognosis by treatment-related endocrine therapy use. Use of these medications was assessed via questionnaires during follow-up (FU) 1 and FU2 for the preceding time interval (median FU time between recruitment and FU1: 6.0 years; median FU time between FU1 and FU2: 5.5 years). Overall, a total of 97% of participants provided information on ever SERM use (yes/no), and 96% of participants provided information on ever AI use (yes/no). If self-reported information on endocrine therapy was not available, data from medical records were used. Examples on SERMs and AIs, as listed in the questionnaires, are included in the supplements.

### Ascertainment of clinical outcomes

Follow-up information from participants was obtained by telephone in 2009 (FU1) and in 2015 (FU2). Information on recurrences, second cancers, and metastatic events were confirmed via medical records or through contact with the treating physicians. Vital status was retrieved through population registries of the study regions up to the end of June 2015, and copies of the death certificates were requested from local health offices. Cause of death was coded according to the 10th revision of the International Classification of Diseases (ICD-10-WHO). The primary outcomes for this study are all-cause mortality, breast cancer (BC)-specific mortality, and risk of recurrence. In the statistical analyses, all-cause mortality was attributed to death by any cause, and BC-specific mortality was attributed to breast cancer (ICD-10-C50) with deaths from other causes censored at date of death. Risk of recurrence in this study corresponds to the outcome “recurrence-free interval” as described in the STEEP criteria [16] including local/regional invasive breast cancer recurrence, metastasis, contralateral disease of the breast, and deaths due to breast cancer. This study includes 438 all-cause deaths, 237 BC-specific deaths, and 376 recurrences. Of the 2282 study participants, four participants were lost to follow-up, and three participants emigrated over the follow-up period.

### Laboratory

Blood samples collected at recruitment have been stored at –80 °C. Median time between diagnosis and blood collection was 3.8 months (range: 0.0, 57.6; eight blood samples collected prior to diagnosis, range: –14.7, –1.6 months). Oxysterol concentrations were measured by blinded personnel in 2,282 blood samples (1,053 EDTA plasma and 1,227 serum), and samples were distributed randomly across study batches. 27-HC (systematic name (25 R),26-hydroxycholesterol) and 25-HC were quantified by Biocrates LifeSciences (Innsbruck, Austria) using a UHPLC-MS/MS with multiple reaction monitoring (MRM) in positive mode using a mass spectrometer with electrospray ionization (ESI). The assay has been validated according to European Medicines Agency (EMA) guidelines. Inter-batch coefficients of variation (CV) were assessed by including 16 blinded replicate quality controls. Mean inter-batch CVs were 15.8% for 27-HC and 29.2% for 25-HC, however 25-HC levels were generally lower in the quality control samples (mean: 12.75 nM) than in the study population (mean: 29.91 nM). Estradiol concentrations were measured using an ELISA in the Division of Cancer Epidemiology laboratory at the German Cancer Research Center (DKFZ) with an inter-batch CV of 16.2%. The ELISA had a sensitivity of 0.03 nM and a standard range of 0.07–7.34 nM.

### Statistical analyses

Biomarker concentrations were log_2_-transformed to normalize the distributions and to allow estimation of the effect of a doubling in concentrations. We detected one outlier for 27-HC and 18 outliers for 25-HC using the Generalized ESD Many-Outlier Procedure [17], and conducted analyses with and without inclusion of outliers. A total of 89 participants had 25-HC levels below the limit of detection (LOD), and 12 participants had estradiol levels below the LOD; we imputed these values with the midpoint between 0 and the lowest detectable value stratified by study region. Spearman partial correlations adjusted for age at diagnosis and study region were used to assess associations between 27-HC, 25-HC, and estradiol, and associations between biomarker concentration and body mass index (BMI) and follow-up time.

We used delayed-entry Cox proportional hazards regression to estimate hazard ratios (HR) and 95% confidence intervals (CI) for risk of all-cause mortality, BC-specific mortality, and risk of recurrence. HR were calculated using biomarker quartiles (higher vs. lowest quartile) and continuous log_2_-transformed values. Tests for trend were calculated modeling the median of the biomarker quartiles as a continuous variable. We used date of diagnosis as start date for follow-up time (time-to-event) and date of blood draw as start date for participants at risk (time-at-risk). Follow-up time ended at the date of the defined outcome (all-cause death, BC-specific death, recurrence) or at the date of last contact or end of follow-up 2 (June 30, 2015), whichever came first. In further analyses, we used a competing-risk model for BC-specific survival; results were similar to those from the models not considering other cause of death as a competing risk. The reverse Kaplan-Meier method was utilized to estimate the median follow-up time for the entire duration of follow-up. We tested the proportional hazards assumption using Schoenfeld residuals and did not observe violation of the proportional hazards assumption.

Established prognostic factors were selected a priori as potential covariates for the survival model: tumor size (≤2 cm, 2–5 cm, >5 cm), nodal status (0, 1–3, >3), and tumor grade (low, moderate, high). All Cox proportional hazard models were additionally adjusted for age at diagnosis, BMI, smoking status (never, former, current smoker), alcohol consumption (0 g/day, <19 g/day, ≥19 g/day), and Charlson Comorbidity Index (CCI), and stratified by ER/ progesterone receptor (PR)-status (ER+ /PR+ , ER+ /PR− or ER− /PR+, ER− /PR− ), and study region (Hamburg, Rhein-Neckar-Karlsruhe). Multicollinearity of covariates was low (variance inflation <1.2). Study participants with missing information for covariates were excluded from the survival analyses (n participants = 17). Endocrine therapy and chemotherapy were not included in the model due to their strong association with hormone-receptor status and tumor characteristics.

Non-parametric restricted cubic splines were used to examine possible non-linearity, comparing models with linear and cubic terms to models with only the linear term [18]. There was no evidence of significant deviation from linearity. We conducted pre-specified subgroup analysis by hormone-receptor status, by median estradiol levels of the cohort (<0.08 nM vs. ≥0.08 nM), and by endocrine therapy: (i) treated with SERM (no AI use), (ii) treated with AI (no SERM use), (iii) treated with both (SERM and AI), and (iv) treated with neither (neither SERM nor AI). We used the binary classification of “ever use” to investigate the effect modification by SERM and AI use due to limited data on use at blood collection. In sensitivity analyses evaluating SERM and AI use at blood collection, we observed small differences in concentrations between self-reported SERM users and non-users (27-HC = 7.4%; 25-HC = 10.2%) and AI users and non-users (27-HC = 0.1%; 25-HC = 12.7%). Statistical heterogeneity of associations between these subgroups was evaluated by including an interaction term into the models.

We evaluated cross-classification of 25-HC and 27-HC at the median concentration using high 27-HC/ low 25-HC as reference due to the observed differential effects of these oxysterols. In sensitivity analyses, we excluded women who died within one year after blood draw (*n* = 22), women who had recurrences within one year after blood draw (*n* = 58), and women whose blood had been collected before breast cancer diagnosis (due to original recruitment as “control”, then re-classification as “case”; *n* = 8). Our primary analyses included the full follow-up period of the cohort. As a separate analysis, we conducted 5-year survival analyses.

All statistical tests were two-tailed and considered significant at *p* < 0.05. Statistical analyses were conducted using SAS 9.4 (SAS Institute Inc., Cary, NC, USA).

## Results

Median age at diagnosis of study participants was 63 years (range: 50–75 years) and median BMI was 25.3 km/m^2^ (range: 15.5–49.6 km/m^2^) (Table 1). Almost all study participants were postmenopausal at diagnosis (91%; remaining participants uncertain menopausal status due to hormone therapy or hysterectomy). A majority of participants (85.9%) had hormone-receptor positive tumors, and 14.2% of participants were hormone-receptor negative. A total of 37.5% of the participants reported exogenous SERM use, 11.2% reported AI use, 32.5% reported use of both a SERM and an AI, and 15.4% of participants reported no endocrine therapy use. Among the no endocrine therapy users, 32.8% had ER/PR-positive and 67.2% ER/PR-negative tumor.

**Table 1:** Baseline characteristics of study population.

Baseline characteristics	n (total)	Median (range) or n (%)
Age at diagnosis (years)	2282	63.0 (50.0-75.0)
BMI (continuous) in kg/m^2^	2279	25.3 (15.5–49.6)
BMI (categories)^a^		
Underweight	35	1.5%
Normal weight	1031	45.2%
Overweight	848	37.2%
Obese	368	16.1%
Study region		
Hamburg	1047	45.9%
Rhein-Neckar-Karlsruhe	1235	54.1%
Median follow-up time (FU2) in years	2282	11.6 (0.3–14.5)
Time between diagnosis and blood draw^b^		
≤3 months	1089	47.7%
>3 months	1193	52.3%
**Tumor characteristics**		
ER/PR status		
ER/PR+	1959	85.9%
ER-/PR-	323	14.2%
HER2/neu status^c^		
HER2+	444	20.6%
HER2-	1717	79.5%
Breast cancer stage at diagnosis		
I	1137	49.8%
IIa	718	31.5%
IIb	273	12.0%
IIIa	154	6.8%
Tumor size		
<2 cm	1401	61.5%
2–5 cm	807	35.4%
>5 cm	72	3.2%
Number of positive lymph nodes		
0	1646	72.1%
1–3	510	22.4%
4–9	126	5.5%
Tumor grade		
Low	469	20.7%
Moderate	1234	54.3%
High	568	25.0%
Endocrine therapy, ever^c^		
SERM^d^	855	37.5%
Aromatase Inhibitor^e^	255	11.2%
Both^f^	742	32.5%
Neither^g^	351	15.4%
Other therapy, ever^c^		
Chemotherapy	1040	46.1%
Radiation therapy	1828	80.8%
27-HC concentrations (nM)	2282	210.0 (85.6–600.0)
25-HC concentrations (nM)^h^	2282	20.4 (2.49–5719.0)
Estradiol concentrations (nM)^i^	2282	0.08 (0.0–4.31)

*BMI* body mass index, *ER* estrogen receptor, *PR* progesterone receptor, *HER2* human epidermal growth factor 2, *SERM* selective estrogen receptor modulator, *27-HC* 27-hydroxychlesterol, *25-HC* 25-hydroxycholesterol, *nM* nanomolar, *LOD* level of detection.

^a^Underweight < 18.5 kg/m^2^; normal weight = 18.5-24.9 kg/m^2^; overweight = 25-29.9 kg/m^2^; obese ≥ 30 kg/m^2^.

^b^Eight participants with blood collection before breast cancer diagnosis (due to original recruitment as “control”, then re-classification as “case”), median = −5.3; range: −14.7, −1.6.

^c^Missing information: Her2-status *n* = 121 (4.3%); endocrine therapy *n* = 79 (3.5%); chemotherapy *n* = 24 (1.1%); radiotherapy *n* = 19 (0.8%).

^d^Only SERM (no aromatase inhibitors).

^e^Only aromatase inhibitors (no SERM).

^f^SERM and aromatase inhibitors use.

^g^Neither SERM nor aromatase inhibitors use.

^h^89 values imputed due to levels below LOD.

^i^12 values imputed due to levels below LOD.

The median follow-up time for the cohort was 11.6 years. Median concentration of 27-HC was 210.0 nM, median concentration of 25-HC was 20.4 nM, and median concentration of estradiol was 0.08 nM (corresponding to 22.2 ng/ml). We observed weak correlations between 27-HC and 25-HC (r = 0.32), and between estradiol and 27-HC and 25-HC (r < |0.1|) (data not shown). Storage time and BMI were weakly correlated with oxysterol concentration (r < |0.16|) (data not shown).

### Associations between 27-HC and breast cancer prognosis

We observed no association between circulating 27-HC and breast cancer outcomes in the overall cohort (Table 2). There was no heterogeneity by tumor hormone receptor-status (all-cause death, p_het_ = 0.76; BC-death, p_het_ = 0.95; recurrences, p_het_ = 0.55).

**Table 2:** Association between circulating 27-HC and breast cancer prognosis—overall, by receptor status, and by estradiol level.

27-HC								
		Q1	Q2	Q3	Q4	p-trend	Per 1 unit increase	P_het_
**All-cause mortality**	**N/Events**							
Overall	2265/434	1.00 (Ref.)	0.86 (0.65,1.14)	1.05 (0.80,1.38)	1.21 (0.93,1.57)	0.07	1.17 (0.89,1.54)	
Hormone receptor status								
ER/PR+	1954/346	1.00 (Ref.)	0.88 (0.65,1.20)	0.99 (0.73,1.35)	1.19 (0.89,1.60)	0.16	1.14 (0.84,1.55)	
ER-/PR-	320/88	1.00 (Ref.)	0.89 (0.44,1.80)	1.55 (0.82,2.94)	1.26 (0.66,2.38)	0.26	1.30 (0.68,2.49)	0.76
Estradiol levels								
<0.08 nM	1132/202	1.00 (Ref.)	1.07 (0.69,1.67)	1.31 (0.87,1.99)	1.60 (1.06,2.41)	0.02	1.80 (1.20,2.71)	
≥0.08 nM	1133/232	1.00 (Ref.)	0.77 (0.53,1.12)	0.94 (0.65,1.36)	0.99 (0.69,1.42)	0.81	0.87 (0.60,1.26)	0.02
**BC-specific mortality**								
Overall	2265/236	1.00 (Ref.)	0.95 (0.65,1.39)	1.02 (0.70,1.49)	1.23 (0.86,1.77)	0.21	1.28 (0.88,1.85)	
Hormone receptor status								
ER/PR+	1945/178	1.00 (Ref.)	0.99 (0.64,1.53)	0.97 (0.63,1.51)	1.30 (0.86,1.96)	0.23	1.33 (0.87,2.04)	
ER-/PR-	320/58	1.00 (Ref.)	1.08 (0.46,2.55)	1.46 (0.65,3.26)	1.35 (0.61,2.95)	0.37	1.36 (0.61,3.00)	0.95
Estradiol levels								
<0.08 nM	1132/117	1.00 (Ref.)	1.06 (0.58,1.97)	1.52 (0.87,2.66)	1.64 (0.95,2.83)	0.04	1.95 (1.14,3.31)	
≥0.08 nM	1133/119	1.00 (Ref.)	0.92 (0.56,1.53)	0.74 (0.43,1.27)	0.96 (0.58,1.59)	0.71	0.88 (0.52,1.49)	0.05
**Recurrences**								
Overall	2265/372	1.00 (Ref.)	0.93 (0.68,1.26)	1.07 (0.79,1.44)	1.27 (0.95,1.70)	0.06	1.30 (0.96,1.76)	
Hormone receptor status								
ER/PR+	1945/287	1.00 (Ref.)	0.97 (0.69,1.37)	0.97 (0.69,1.36)	1.24 (0.89,1.73)	0.21	1.25 (0.89,1.76)	
ER-/PR-	320/85	1.00 (Ref.)	0.91 (0.43,1.93)	1.56 (0.81,3.01)	1.46 (0.76,2.80)	0.12	1.49 (0.77,2.88)	0.55
Estradiol levels								
<0.08 nM	1132/188	1.00 (Ref.)	0.89 (0.57,1.41)	1.11 (0.73,1.68)	1.19 (0.78,1.80)	0.27	1.43 (0.92,2.20)	
≥0.08 nM	1133/184	1.00 (Ref.)	1.07 (0.70,1.65)	1.09 (0.70,1.70)	1.38 (0.91,2.10)	0.14	1.21 (0.79,1.85)	0.73

Hazard ratios (HR) and 95% confidence intervals (95% CI) from delayed-entry Cox proportional hazard models. All models are adjusted for age at diagnosis, prognostic factors (tumor size, nodal status, histological grading), BMI, smoking status, alcohol consumption, Charlson Comorbidity Index, and stratified by study region and ER/PR-status. p_trend_ for the median of quartiles. Quartiles were calculated using the center-specific distribution. A 1-unit increase in the log_2_ transformed 27-HC concentration corresponds to a doubling. P_het_ comparing hormone-receptor positive vs. negative subgroup or low vs. high estradiol subgroup. n participants with missing covariate information = 17.

*BC* breast cancer, *27-HC* 27-hydroxycholesterol, *nM* nanomolar, *ER* estrogen receptor, *PR* progesterone receptor.

We observed heterogeneity in associations between 27-HC and all-cause death (p_het_ = 0.02) and BC-specific death (p_het_ = 0.05) by circulating estradiol levels (below vs. above median). Among women in the low estradiol subgroup, a doubling of 27-HC concentration was associated with higher risk of all-cause death (HR_log2_ = 1.80 (1.20–2.71), p_trend_ = 0.02) and BC-specific death (HR_log2_ = 1.95 (1.14–3.31), p_trend_ = 0.04), whereas no significant associations were observed among women in the high estradiol subgroup.

We next investigated the association between 27-HC and breast cancer prognosis in subgroups stratified by endocrine therapy (Table 3). Among those participants not using endocrine therapy, higher 27-HC levels were associated with a higher risk of recurrences (HR_log2_ = 2.42 (1.11–5.28)); when further stratifying non-endocrine therapy users, the effect was only observed in hormone-receptor negative participants (all cause-death, HR_log2_ = 3.09 (1.23–7.76); BC-specific death, HR_log2_ = 3.96 (1.23–12.77); recurrences, HR_log2_ = 3.50 (1.42–8.62)), and in low-estradiol participants (all-cause death, HR_log2_ = 4.42 (1.28-15.24); BC-specific death, HR_log2_ = 5.27 (1.19–23.25); recurrences, HR_log2_ = 3.82 (1.25–11.64)). Furthermore, higher 27-HC levels were associated with a poorer prognosis among SERM users (BC-specific death, HR_log2_ = 2.29 (1.12-4.71); recurrences, HR_log2_ = 2.30 (1.28-4.14)), while no significant association was observed among AI users.

**Table 3:** Association between circulating 27-HC and breast cancer prognosis—by endocrine therapy.

27-HC												
	SERM			AI			Both			None		
	N/Events	HR (95% CI), per 1 unit increase	p_trend_	N/Events	HR (95% CI), per 1 unit increase	p_trend_	N/Events	HR (95% CI), per 1 unit increase	p_trend_	N/Events	HR (95% CI), per 1 unit increase	p_trend_
**All-cause mortality**												
Overall	851/158	1.42 (0.90,2.25)	0.16	250/54	0.54 (0.23,1.29)	0.25	739/108	1.33 (0.74,2.38)	0.27	346/65	1.27 (0.61,2.62)	0.11
Hormone receptor status												
ER/PR+	826/152	1.57 (0.98,2.51)	0.07	245/52	0.65 (0.27,1.52)	0.46	722/102	1.38 (0.77,2.50)	0.24	112/19	0.30 (0.06,1.45)	0.51
ER-/PR-	25/6	**–**		5/2	**–**		17/6	**–**		234/46	3.09 (1.23,7.76)	0.01
Estradiol levels												
<0.08 nM	389/67	2.06 (0.95,4.49)	0.24	135/25	0.79 (0.23,2.69)	0.67	385/52	1.77 (0.73,4.32)	0.21	188/36	4.42 (1.28,15.24)	0.05
≥0.08 nM	462/91	1.20 (0.66,2.16)	0.47	115/29	0.26 (0.07,0.95)	0.08	354/56	1.16 (0.51,2.61)	0.73	158/29	0.52 (0.17,1.53)	0.97
**BC-specific mortality**												
Overall	851/69	2.29 (1.12,4.71)	0.10	250/27	0.37 (0.10,1.40)	0.25	739/73	1.25 (0.61,2.58)	0.44	346/40	2.30 (0.85,6.22)	0.09
Hormone receptor status												
ER/PR+	826/67	2.54 (1.22,5.28)	0.06	245/25	0.52 (0.14,1.93)	0.50	722/69	1.26 (0.61,2.58)	0.41	112/9	0.16 (0.01,2.41)	0.53
ER-/PR-	25/2	–		5/2	**–**		17/4	**–**		234/31	3.96 (1.23,12.77)	0.02
Estradiol levels												
<0.08 nM	389/31	4.25 (1.31,13.78)	0.08	135/11	0.52 (0.00,86.58)	0.73	385/36	1.67 (0.57,4.85)	0.43	188/24	5.27 (1.19,23.25)	0.07
≥0.08 nM	462/38	1.58 (0.60,4.21)	0.68	115/16	0.10 (0.01,1.36)	0.14	354/37	1.09 (0.37,3.19)	0.65	158/16	0.96 (0.17,5.29)	0.58
**Recurrences**												
Overall	851/108	2.30 (1.28,4.14)	0.04	250/41	0.64 (0.23,1.79)	0.57	739/130	0.85 (0.51,1.43)	0.61	346/61	2.42 (1.11,5.28)	0.01
Hormone receptor status												
ER/PR+	826/101	2.52 (1.38,4.62)	0.02	245/39	0.70 (0.25,1.94)	0.57	722/123	0.85 (0.50,1.44)	0.62	112/14	0.41 (0.05,3.69)	0.81
ER-/PR-	25/7	–		5/2	**–**		17/7	**–**		234/47	3.50 (1.42,8.62)	0.003
Estradiol levels												
<0.08 nM	389/45	2.30 (0.86,6.13)	0.51	135/18	1.88 (0.40,8.94)	0.69	385/72	1.00 (0.47,2.13)	0.75	188/37	3.82 (1.25,11.64)	0.02
≥0.08 nM	462/63	2.35 (1.09,5.06)	0.05	115/23	0.22 (0.03,1.53)	0.19	354/58	0.95 (0.43,2.10)	0.73	158/24	1.21 (0.32,4.55)	0.32

Hazard ratios (HR) and 95% confidence intervals (95% CI) from delayed-entry Cox proportional hazard models. All models are adjusted for age at diagnosis, prognostic factors (tumor size, nodal status, histological grading), BMI, smoking status, alcohol consumption, Charlson Comorbidity Index, and stratified by study region and ER/PR-status. A 1-unit increase in the log_2_ transformed 27-HC concentration corresponds to a doubling. SERM = only SERM use (no AI); AI = only AI use (no SERM); Both = SERM and AI use; None = neither SERM nor AI use. n participants with missing covariate information = 17.

*BC* breast cancer, *27-HC* 27-hydroxycholesterol, *nM* nanomolar, *ER* estrogen receptor, *PR* progesterone receptor, *SERM* selective estrogen receptor modulator, *AI* aromatase inhibitor.

### Association between 25-HC and breast cancer prognosis

Higher concentrations of 25-HC were associated with a lower risk of recurrences (HR_log2_ = 0.87 (0.77–0.98), p_trend_ = 0.19) in the overall cohort (Table 4); no association was observed for all-cause death or BC-specific death.

**Table 4:** Association between circulating 25-HC and breast cancer prognosis – overall, by receptor status, and by estradiol level.

25-HC								
		Q1	Q2	Q3	Q4	p-trend	Per 1 unit increase	P_het_
**All-cause mortality**	**N/Events**							
Overall	2265/434	1.00 (Ref.)	0.99 (0.75,1.31)	0.97 (0.73,1.28)	1.07 (0.81,1.40)	0.69	1.00 (0.89,1.12)	
Hormone receptor status								
ER/PR+	1954/346	1.00 (Ref.)	1.10 (0.81,1.51)	1.00 (0.73,1.37)	1.20 (0.88,1.63)	0.35	1.04 (0.91,1.18)	
ER-/PR-	320/88	1.00 (Ref.)	0.82 (0.45,1.48)	0.92 (0.50,1.69)	0.64 (0.33,1.25)	0.28	0.79 (0.58,1.07)	0.05
Estradiol levels								
<0.08 nM	1132/202	1.00 (Ref.)	1.04 (0.69,1.57)	0.88 (0.58,1.33)	1.38 (0.94,2.04)	0.19	1.08 (0.91,1.28)	
≥0.08 nM	1133/232	1.00 (Ref.)	0.92 (0.62,1.35)	1.02 (0.69,1.49)	0.83 (0.56,1.22)	0.46	0.94 (0.80,1.10)	0.54
**BC-specific mortality**								
Overall	2265/236	1.00 (Ref.)	0.97 (0.67,1.41)	0.97 (0.67,1.41)	1.00 (0.69,1.45)	1.00	0.94 (0.80,1.10)	
Hormone receptor status								
ER/PR+	1945/178	1.00 (Ref.)	1.18 (0.76,1.82)	1.07 (0.69,1.65)	1.10 (0.71,1.69)	0.82	0.96 (0.81,1.15)	
ER-/PR-	320/58	1.00 (Ref.)	0.68 (0.33,1.44)	0.84 (0.39,1.78)	0.79 (0.36,1.71)	0.79	0.79 (0.54,1.15)	0.28
Estradiol levels								
<0.08 nM	1132/117	1.00 (Ref.)	1.01 (0.57,1.78)	1.02 (0.59,1.77)	1.67 (1.00,2.78)	0.05	1.11 (0.89,1.37)	
≥0.08 nM	1133/119	1.00 (Ref.)	0.87 (0.52,1.46)	0.93 (0.55,1.57)	0.57 (0.32,1.00)	0.07	0.81 (0.66,1.01)	0.05
**Recurrences**								
Overall	2265/372	1.00 (Ref.)	0.84 (0.62,1.13)	0.88 (0.66,1.17)	0.80 (0.60,1.07)	0.19	0.87 (0.77,0.98)	
Hormone receptor status								
ER/PR+	1945/287	1.00 (Ref.)	0.88 (0.63,1.22)	0.83 (0.60,1.15)	0.74 (0.53,1.04)	0.08	0.87 (0.76,0.98)	
ER-/PR-	320/85	1.00 (Ref.)	0.77 (0.41,1.47)	1.02 (0.54,1.94)	0.94 (0.50,1.80)	0.89	0.85 (0.61,1.16)	0.88
Estradiol levels								
<0.08 nM	1132/188	1.00 (Ref.)	0.92 (0.60,1.40)	0.94 (0.62,1.41)	1.03 (0.69,1.55)	0.87	0.91 (0.77,1.07)	
≥0.08 nM	1133/184	1.00 (Ref.)	0.75 (0.49,1.14)	0.85 (0.56,1.31)	0.64 (0.41,0.98)	0.09	0.86 (0.72,1.02)	0.51

Hazard ratios (HR) and 95% confidence intervals (95% CI) from delayed-entry Cox proportional hazard models. All models are adjusted for age at diagnosis, prognostic factors (tumor size, nodal status, histological grading), BMI, smoking status, alcohol consumption, Charlson Comorbidity Index, and stratified by study region and ER/PR-status. p_trend_ for the median of quartiles. Quartiles were calculated using the center-specific distribution. A 1-unit increase in the log_2_ transformed 25-HC concentration corresponds to a doubling. P_het_ comparing hormone-receptor positive vs. negative subgroup or low vs. high estradiol subgroup. n participants with missing covariate information = 17.

*BC* breast cancer, *25-HC* 25-hydroxycholesterol, *nM* nanomolar, *ER* estrogen receptor, *PR* progesterone receptor.

We observed no heterogeneity in associations by hormone receptor status, but by circulating estradiol levels (below vs. above median; BC-specific death p_het_ = 0.05). In participants with low estradiol levels, 25-HC in the highest quartile was associated with a higher risk of BC-specific death (HR_Q4vs.Q1_ = 1.67 (1.00–2.78)) as compared to the lowest quartile, while a better prognosis was observed in participants with higher estradiol levels (HR_Q4vs.Q1_ = 0.57 (0.32–1.00)).

In subgroup analyses stratified by endocrine therapy (Table 5), we observed that higher 25-HC levels were associated with a lower risk of recurrences among SERM users (HR_log2_ = 0.78 (0.63–0.97)), and among those participant who were using both SERM and AI (HR_log2_ = 0.76 (0.61–0.95). No associations were observed for AI users or non-endocrine therapy users. However, in participants not using endocrine therapy, we observed significant interactions in associations by estradiol levels (BC-specific death, lower estradiol levels HR_log2_ = 1.54 (0.82-2.88), p_trend_ = 0.03; higher estradiol levels HR_log2_ = 0.50 (0.27–0.95), p_trend_ = 0.07).

**Table 5:** Association between circulating 25-HC and breast cancer prognosis – by endocrine therapy.

25-HC												
	SERM			AI			Both			None		
	N/Events	HR (95% CI), per 1 unit increase	p_trend_	N/Events	HR (95% CI), per 1 unit increase	p_trend_	N/Events	HR (95% CI), per 1 unit increase	p_trend_	N/Events	HR (95% CI), per 1 unit increase	p_trend_
**All-cause mortality**												
Overall	851/158	1.05 (0.86,1.28)	0.80	250/54	1.06 (0.75,1.49)	0.41	739/108	0.89 (0.70,1.14)	0.54	346/65	1.03 (0.70,1.51)	0.45
Hormone receptor status												
ER/PR+	826/152	1.07 (0.87,1.30)	0.64	245/52	1.08 (0.77,1.50)	0.31	722/102	0.92 (0.72,1.17)	0.81	112/19	1.56 (0.72,3.39)	0.48
ER-/PR-	25/6	**–**		5/2	**–**		17/6	**–**		234/46	0.95 (0.58,1.55)	0.48
Estradiol levels												
<0.08 nM	389/67	0.95 (0.71,1.27)	0.35	135/25	1.03 (0.59,1.79)	0.59	385/52	0.95 (0.65,1.39)	0.99	188/36	1.39 (0.82,2.35)	0.05
≥0.08 nM	462/91	1.22 (0.90,1.65)	0.23	115/29	1.08 (0.70,1.66)	1.00	354/56	0.80 (0.57,1.11)	0.28	158/29	0.70 (0.41,1.19)	0.16
**BC-specific mortality**												
Overall	851/69	0.84 (0.64,1.12)	0.31	250/27	0.93 (0.57,1.50)	0.79	739/73	0.94 (0.68,1.28)	0.88	346/40	0.94 (0.59,1.49)	0.33
Hormone receptor status												
ER/PR+	826/67	0.87 (0.66,1.15)	0.39	245/25	0.99 (0.62,1.57)	0.55	722/69	0.97 (0.72,1.31)	0.58	112/9	1.09 (0.43,2.80)	0.98
ER-/PR-	25/2	**–**		5/2	**–**		17/4	**–**		234/31	0.89 (0.51,1.54)	0.60
Estradiol levels												
<0.08 nM	389/31	0.80 (0.54,1.20)	0.28	135/11	0.35 (0.07,1.69)	0.63	385/36	1.00 (0.61,1.64)	0.55	188/24	1.54 (0.82,2.88)	0.03
≥0.08 nM	462/38	0.99 (0.66,1.49)	0.68	115/16	0.92 (0.44,1.93)	0.93	354/37	0.79 (0.51,1.24)	0.39	158/16	0.50 (0.27,0.95)	0.07
**Recurrences**												
Overall	851/108	0.78 (0.63,0.97)	0.06	250/41	1.07 (0.73,1.55)	0.85	739/130	0.76 (0.61,0.95)	0.08	346/61	0.95 (0.66,1.37)	0.98
Hormone receptor status												
ER/PR+	826/101	0.79 (0.64,0.98)	0.07	245/39	1.15 (0.82,1.61)	0.73	722/123	0.77 (0.62,0.95)	0.15	112/14	1.00 (0.54,1.86)	0.50
ER-/PR-	25/7	–		5/2	–		17/7	–		234/47	0.96 (0.59,1.54)	0.22
Estradiol levels												
<0.08 nM	389/45	0.82 (0.60,1.13)	0.39	135/18	1.08 (0.54,2.18)	0.80	385/72	0.71 (0.52,0.98)	0.29	188/37	1.12 (0.68,1.85)	0.10
≥0.08 nM	462/63	0.81 (0.62,1.08)	0.12	115/23	1.61 (0.86,3.00)	0.90	354/58	0.82 (0.61,1.11)	0.41	158/24	0.67 (0.37,1.21)	0.45

Hazard ratios (HR) and 95% confidence intervals (95% CI) from delayed-entry Cox proportional hazard models. All models are adjusted for age at diagnosis, prognostic factors (tumor size, nodal status, histological grading), BMI, smoking status, alcohol consumption, Charlson Comorbidity Index, and stratified by study region and ER/PR-status. A 1-unit increase in the log_2_ transformed 27-HC concentration corresponds to a doubling. SERM = only SERM use (no AI); AI = only AI use (no SERM); Both = SERM and AI use; None = neither SERM nor AI use. n participants with missing covariate information = 17.

*BC* breast cancer, *25-HC* 25-hydroxycholesterol, *nM* nanomolar, *ER* estrogen receptor, *PR* progesterone receptor, *SERM* selective estrogen receptor modulator, *AI* aromatase inhibitor.

### Additional analyses

Additional adjustment for estradiol concentration and biomarker concentration (i.e. 27-HC models additionally adjusted for 25-HC concentrations, and vice versa), mode of detection, chemotherapy, and endocrine therapy (SERM and AI) had minimal impact on the effect estimates.

In exploratory analyses of 27-HC and 25-HC cross-classified, we observed that participants with high 27-HC/low 25-HC levels had a significantly higher risk of recurrences as compared to participants with low 27-HC/high 25-HC (HR = 1.65 (1.11–2.46)) (Table S1). No statistically significant results were observed for the high/high and low/low group.

The associations for the 5-year survival analysis between 27-HC, 25-HC, and breast cancer prognosis were similar to the associations of the main analyses (Table S2 and Table S3).

Excluding participants with recurrences within one year after blood draw attenuated the association between 27-HC and risk of recurrence (from HR = 1.30 (0.94–1.74) to HR = 1.16 (0.84–1.62)). Exclusion of outliers, women who died within one year after blood draw and women whose blood had been collection before breast cancer diagnosis, did not meaningfully change the risk estimates for any of the outcomes (<10%) (data not shown).

## Discussion

We assessed associations between 27-HC, 25-HC, and survival after a breast cancer diagnosis in a well-characterized cohort of 2282 breast cancer patients, observing no association between 27-HC and breast cancer survival overall, while higher levels of 25-HC were associated with a lower risk of recurrence. Higher levels of 27-HC were associated with a poorer prognosis among participants with relatively low estradiol levels and among participants not using endocrine therapy. The findings of the main analyses were supported by the 5-year survival analyses reporting similar effect estimates. Our study aims were based on strong experimental data for 27-HC and breast cancer development and progression and a previous epidemiological study associating 27-HC with breast cancer risk. We investigated the outcomes of all-cause mortality and BC-specific mortality given that (1) 27-HC is suggested to have a role in multiple physiologic processes and (2), 27-HC binds to the estrogen receptor in tissues beyond breast cancer; evaluations stratified by estradiol were conducted given previous experimental and other epidemiological studies suggesting actions of oxysterols may depend on the background estrogenic environment.

27-HC is the most extensively studied oxysterol with respect to breast cancer. It was identified as the first endogenous SERM, exhibiting agonistic or antagonistic effects depending on the target tissue [19]. 25-HC, which is structurally similar to 27-HC, is an ER modulator and was reported to have similar, but weaker, agonist activities to 27-HC [7]. In vitro models demonstrated that 27-HC administration resulted in increased ER+ breast cancer growth and proliferation [9, 20]. Similar effects were observed in in vivo studies [8, 9]. It is hypothesized that 27-HC may act as a partial agonist in breast cells [7, 20]: In patients with low estradiol levels, 27-HC is able to bind to the ER exerting weak estrogenic actions, and consequently cell proliferation; in patients with high estradiol levels, on the other hand, 27-HC may be able to reduce the proliferative actions of estradiol through competitive binding. This hypothesis is supported by reports of different levels of potency of 27-HC and estradiol on the ER. For 27-HC, prior studies have reported binding affinity (K_i_) at 1.32 µM (=1320 nM) for the ERα and 0.42 µM (= 420 nM) for ERβ [7], in comparison to values (K_d_) of 0.1 nM (ERα) and 0.4 nM (ERβ) for estradiol [21]. Using a Gal4-ER cotransfection assay, it was shown that 27-HC and 25-HC significantly inhibited estradiol activation of ERα and ERβ in the presence of 0.5 nM estradiol with 27-HC being the most potent inhibitor (half-maximal inhibitory concentration, IC_50_ = 1 µM) [7]. DuSell et al. demonstrated that increasing levels of 27-HC reduced estradiol-induced transcriptional activity of ERα at physiological relevant estradiol levels of 0.5 nM [6]. As circulating levels of 27-HC in this study are approximately 200 nM, with maximum values of 600 nM, and median circulating estradiol levels are 0.08 nM, the hypothesis of competitive [7] or allosteric binding [22] of 27-HC would be physiological plausible. The interactions between these oxysterols and estradiol in vivo need to be further characterized in experimental models to allow a better understanding of the relative contributions of each of these analytes on the ER-mediated signaling pathways.

Clinical and epidemiologic studies on circulating oxysterols and breast cancer progression are sparse. To our knowledge, only one study directly assessed circulating free oxysterols (*n* = 58 breast cancer patients), and reported, similar to our overall findings, no significant association between plasma levels of 27-HC, 25-HC, and disease-free survival [14]. This prior study evaluated free oxysterol concentrations as compared to “total” oxysterols (free and esterified) in our study; free and total 27-HC levels (*r* = 0.63) and free and total 25-HC (*r* = 0.54) are modestly correlated [23]. In a prior study of our working group, higher circulating levels of 27-HC were inversely associated with breast cancer risk among postmenopausal participants (RR_Q4vsQ1_ = 0.56 (0.36–0.87)) [10]. Larger epidemiological studies evaluating tumor tissue samples have indirectly investigated the effect of 27-HC on breast cancer prognosis by quantifying the levels of protein or mRNA expression of the enzymes converting cholesterol to 27-HC (CYP27A1) or metabolizing 27-HC to downstream metabolites (CYP7B1). Two studies reported that CYP27A1 was associated with better recurrence-free survival in pre-menopausal women (HR_protein_ = 0.42 (0.21–0.84); HR_mRNA_ = 0.38 (0.18–0.78)) [12] and better recurrence-free survival (HR = 0.60 (0.39–0.90)) and overall survival (HR = 0.41 (0.23-0.72)) in patients with ERα-positive breast cancer and ≤50 years [11], while another study from Kimbung et al. reported a poorer long-term prognosis with high CYP27A expression (overall survival, HR = 1.89 (1.25–2.85); BC-specific survival, HR = 2.25 (1.26–4.01)) in a population including 90% of breast cancer patients ≥55 years [13]. No significant correlation for the expression of CYP27A1 mRNA and survival was observed in a study of Nelson et al. [8]. Two studies assessing CYP7B1, the enzyme metabolizing 27-HC, reported similar findings observing that higher CYP7B1 expression was associated with better survival in hormone-receptor positive breast cancer patients [8, 9]. CYP7B1 metabolizes 27-HC to its downstream products, suggesting a higher clearance of 27-HC. In summary, the findings of prior human studies suggest a potential dual role for 27-HC in breast cancer progression as higher CYP27A1, the enzyme converting 27-HC, expression was generally associated with a poorer prognosis among postmenopausal participants [13] and a better prognosis among pre-menopausal [12] or presumably pre-menopausal breast cancer patients [11]. Further data suggest 27-HC may act as a negative allosteric modifier (i.e., modifying receptor response) rather than a classical competitive agonist to estradiol [22]. The findings of this study support the previous reports of a potential dual role of 27-HC on breast cancer progression depending on the estradiol levels. Higher levels of 27-HC were associated with a poorer prognosis among participants in the low estradiol subgroup, while no significant associations were observed in the high-estradiol subgroup.

We observed no heterogeneity in associations by hormone-receptor status. Beyond the ER-mediated pathway, another pathway linking oxysterols to breast cancer progression includes the liver X receptor (LXR), primarily expressed in liver, intestine, adipocytes, and macrophages with various functions including the regulation of the cholesterol metabolism. Oxysterols including 27-HC and 25-HC, but with lower potency, were reported to be the main ligands to the LXR [24, 25]. It was suggested that the LXR can, similar to the ER, exert differential activities in a context-specific manner [26, 27], for example 27-HC was reported to selectively modulate immune suppression via the LXR [28–30]. Experimental studies reported reduced cell-proliferation in ER-positive breast cancer cells after LXR ligand treatment [31, 32], which may explain the protective effects observed in this study. On the other hand, LXR activation was reported to convey chemotherapy resistance in triple-negative breast cancer [33], potentially explaining the observed poorer prognosis in hormone-receptor negative breast cancer patient and participants not treated with endocrine therapy. Activation of LXR signaling was reported for low concentrations of 27-HC in prior experimental studies on myeloid immune cells [30] and ovarian cancer cells [34]. In a prior study on circulating 27-HC and breast cancer risk including 287 women from the EPIC Heidelberg cohort, no heterogeneity by LXR-β expression in tumor tissue among postmenopausal women was observed [35]. However, the physiological dose in vivo required for the LXR mechanism to be relevant in breast cancer progression in women remains to be established.

In further subgroup analyses, we assessed associations between 27-HC, 25-HC, and breast cancer prognosis by endocrine therapy use due to a potential interaction between the endogenous ER modulators 27-HC and 25-HC and endocrine therapy. SERMs are synthetic drugs that bind to the ER and exert estrogenic or anti-estrogenic effects depending on the target tissues. AI, mainly used in postmenopausal patients, block aromatase action, the enzyme synthesizing estrogen, resulting in lower systemic estrogen levels. While higher levels of 27-HC were generally associated with a poorer prognosis among SERM users, 25-HC levels were associated with a better prognosis in SERM users. Furthermore, we observed effect heterogeneity by estradiol levels among the no-endocrine therapy participants with a poorer prognosis in the low-estradiol subgroup and a better prognosis in the higher estradiol subgroup for higher 27-HC and 25-HC levels.

The study of Dalenc et al. reported increased 27-HC levels after AI treatment and decreased 25-HC levels after tamoxifen treatment over 28 days, however the magnitude of association was not fully described [36]. We stratified our analyses by reported ever use of endocrine therapies given the weak associations between these therapies and circulating 27-HC and 25-HC levels, and that use of these therapies at any time over follow-up would potentially impact the associations between 27-HC or 25-HC and breast cancer progression. Nevertheless, findings by endocrine therapy should be interpreted with caution.

Lastly, it needs to be noted that 27-HC plays a role in a number of other mechanisms including: reduction of P53 transcriptional activity [37], production of reactive oxygen species (ROS) leading to STAT3 activation, and VEGF signaling resulting in induced angiogenesis [38, 39], induction of epithelial-mesenchymal transition (EMT) [39, 40], and secretion of chemokines enhancing the production of macrophages which in turn further produce 27-HC [39]. 27-HC is also a ligand to several other receptors including sterol regulatory element binding protein (SREBP), peroxisome proliferator-activated receptors (PPARs), retinoic acid receptor related orphan receptor (ROR), toll like receptors (TLRs), and insulin-induced gene 1 protein (INSIG) [41, 42], leaving the possibility that other -previously undetected- mechanisms may have an impact on the observed associations.

We provide novel data on the association between circulating levels of the structurally similar oxysterols 27-HC, 25-HC, and breast cancer prognosis in a study with detailed data on disease characteristics, treatment characteristics and outcomes. Importantly, we were able to account for estradiol levels and endocrine therapy use in our analyses. Blood samples were collected non-fasting in this study; however, the impact of fasting status on 27-HC concentration was demonstrated to be relatively weak (fasting status at blood draw ≥3 h vs. <3 h, percent difference = −2.79, *p* trend = 0.08) [43]. A limitation of the study is the missing information on blood cholesterol levels. Lu et al. reported that the associations between 27-HC and breast cancer risk were attenuated after additionally adjusting for total cholesterol levels, however, adjusting for both cholesterol and estrone levels resulted in the same associations as using the unadjusted model [10]. In addition, total cholesterol was reported to be only weakly correlated with post-diagnosis 27-HC (r = 0.38) [11] and moderately correlated with pre-diagnosis 27-HC (*r* = 0.43) [10]. Data were not available to allow evaluation of associations by use of cholesterol-lowering medications such as statins. A prior study from our research group [43] and others [44] did not observe differences in 27-HC concentrations by statin use. While a study of Kimbung et al. reported a decrease in 27-HC levels following statin treatment, the change in 27-HC levels was not associated with an anti-proliferative effect in tumors [11]. Furthermore, we were limited to evaluating ever SERM and AI use, as we did not have information on the exact start and end of endocrine therapy use. Thus, associations by endocrine therapy need to be interpreted in that context.

Finally, the CV of 25-HC was 29.2%, potentially resulting in exposure misclassification; however, it merits noting that the concentrations in the quality control samples were substantially lower than those in the study samples, and the CVs may not reflect the precision of the assay at higher analyte concentrations. Although, to our knowledge, decomposition of oxysterols at very low temperatures has not been reported, we cannot rule out the possibility that long-term storage could have had an effect on the sample concentrations [45, 46].

Lastly, while the current study was hypothesis based, we carried out multiple analyses to examine associations and did not adjust the significance level for multiple testing. Thus, it is possible that some of our findings are due to chance.

We provide the first data on circulating 27-HC, 25-HC, and breast cancer outcomes in a large breast cancer cohort suggesting differential associations between 27-HC, 25-HC, and prognosis in breast cancer patients depending on the underlying estrogenic environment. For a better characterization of the potential importance of these cholesterol metabolites in women with a breast cancer diagnosis further experimental studies better describing 27-HC, 25-HC, and estradiol and their overlapping and independent signaling pathways in breast cancer are required, together with epidemiological studies assessing 27-HC and 25-HC by estradiol levels and endocrine therapy.

## Supplementary information

Supplemental material

## Data Availability

The datasets generated and analyzed during the current study are available from the corresponding author on reasonable request.
