# Supplementary material for: Endogenous estrogen receptor modulating oxysterols and breast cancer prognosis: Results from the MARIE patient cohort
Source: Br J Cancer. 2023 Jun 24;129(3):492–502. doi: 10.1038/s41416-023-02315-w (PMC10403581; doi:10.1038/s41416-023-02315-w)
Supplement: Supplementary file 1 — Supplemental material [file 41416_2023_2315_MOESM1_ESM.docx]

# Supplementary information

### Endocrine therapy:

The category “SERMs” includes treatment with exogenous SERMs (e.g., Tamoxifen, Mandofen, Nolvadex, Tamokadin, Tamox, Tamox-GRY, Tamoxifen AbZ, Tamoxifen beta, Tamoxifen-CT, Tamoxifen NC, Tamoxifen-ratiopharm, Tamoxistad, Valodex, Istubal). Six women reported treatment with Raloxifen (e.g., Evista, Optruma); they were included in the “SERMs” category. The category “aromatase inhibitors” (AI) includes, for example, Anastrazol (Arimidex), Letrozol (Femara), Exemestan (Aromasin), Aminogluthemid (Orimeten).

### Table S1: Association between circulating 27-HC, 25-HC, and breast cancer prognosis: Cross-classified

| Cross-classification | | | | | | | |
| --- | --- | --- | --- | --- | --- | --- | --- |
|  | **Reference:  low 27-HC/high 25-HC** | **high 27-HC/low 25-HC** | | **low 27-HC/low 25-HC** | | **high 27-HC/high 25-HC** | |
|  | **n/events** | **n/events** | **HR (95% CI)** | **n/events** | **HR (95% CI)** | **n/events** | **HR (95% CI)** |
| All-cause mortality |  |  |  |  |  |  |  |
| Overall | 457/90 | 461/92 | 1.16 (0.81,1.67) | 674/113 | 0.90 (0.66,1.22) | 672/139 | 1.10 (0.83,1.45) |
| ER/PR+ | 410/78 | 384/67 | 0.95 (0.65,1.39) | 581/89 | 0.84 (0.60,1.18) | 569/112 | 1.08 (0.80,1.46) |
| ER-/PR- | 47/12 | 77/25 | **4.78 (1.74,13.09)** | 93/24 | 1.01 (0.45,2.27) | 103/28 | 1.55 (0.67,3.62) |
| BC-specific mortality | |  |  |  |  |  |  |
| Overall | 457/49 | 461/55 | 1.35 (0.84,2.15) | 674/61 | 0.89 (0.59,1.36) | 672/71 | 0.91 (0.62,1.33) |
| ER+/PR+ | 410/41 | 384/40 | 1.21 (0.73, 2.02) | 581/44 | 0.79 (0.48,1.28) | 569/53 | 0.89 (0.58,1.36) |
| ER-/PR- | 47/8 | 77/15 | **7.93 (2.15,29.30)** | 93/17 | 1.36 (0.51,3.64) | 103/18 | 1.46 (0.51,4.16) |
| Recurrence |  |  |  |  |  |  |  |
| Overall | 457/71 | 461/93 | **1.65 (1.11,2.46)** | 672/100 | 1.15 (0.81,1.63) | 665/109 | 1.01 (0.74,1.39) |
| ER+/PR+ | 410/59 | 384/68 | **1.17 (0.77,1.79)** | 579/80 | 1.11 (0.76,1.64) | 563/81 | 0.97 (0.68,1.37) |
| ER-/PR- | 47/12 | 77/25 | **4.72 (1.61,13.80)** | 93/20 | 1.12 (0.47,2.68) | 102/29 | 1.40 (0.62,3.16) |

Hazard ratios (HR) and 95% confidence intervals (95% CI) from delayed-entry Cox proportional hazard models. All models are adjusted for age at diagnosis, prognostic factors (tumor size, nodal status, histological grading), BMI, smoking status, alcohol consumption, Charlson Comorbidity Index, and stratified by study region and ER/PR-status. HR per 1-unit increase (a 1-unit increase in the log_2_ transformed concentration corresponds to a doubling).
Abbreviations: BC=breast cancer; 27-HC=27-hydroxycholesterol; 25-HC=25-hydroxycholesterol; ER=estrogen receptor; PR=progesterone receptor

### Table S2: Association between circulating 27-HC and breast cancer prognosis: 5-year survival

| 27-HC | | | | | | |  |
| --- | --- | --- | --- | --- | --- | --- | --- |
|  | **Overall** | | **Estradiol < 0.08 nM** | | **Estradiol ≥ 0.08 nM** | |  |
|  | **n/events** | **HR (95% CI),  per 1 unit increase** | **n/events** | **HR (95% CI),  per 1 unit increase** | **n/events** | **HR (95% CI),  per 1 unit increase** | **P_het_ (estradiol)** |
| All-cause mortality |  |  |  |  |  |  |  |
| Overall | 2265/139 | 0.91 (0.57,1.46) | 1132/62 | 1.89 (0.92,3.85) | 1133/77 | **0.50 (0.26,0.96)** | **0.003** |
| ER/PR+ | 1945/94 | 0.78 (0.45,1.38) | 954/35 | 1.91 (0.76,4.79) | 991/59 | **0.45 (0.22,0.94)** | **0.01** |
| ER-/PR- | 320/45 | 1.28 (0.55,2.97) | 178/27 | 1.32 (0.42,4.17) | 143/18 | 1.99 (0.38,10.51) | 0.73 |
| BC-specific mortality |  |  |  |  |  |  |  |
| Overall | 2265/87 | 1.05 (0.57,1.94) | 1132/40 | 1.41 (0.57,3.48) | 1133/47 | 0.82 (0.35,1.90) | 0.31 |
| ER/PR+ | 1945/50 | 1.09 (0.48,2.47) | 954/19 | 1.25 (0.35,4.39) | 991/31 | 0.89 (0.31,2.56) | 0.60 |
| ER-/PR- | 320/37 | 1.11 (0.45,2.78) | 178/21 | 1.16 (0.32,4.18) | 142/16 | 1.64 (0.28,9.67) | 0.66 |
| Recurrence | |  |  |  |  |  |  |
| Overall | 2265/90 | 1.17 (0.64,2.14) | 1132/41 | 1.41 (0.57,3.48) | 1133/49 | 0.91 (0.40,2.07) | 0.32 |
| ER/PR+ | 1945/51 | 1.15 (0.51,2.58) | 954/19 | 1.25 (0.35,4.39) | 991/32 | 0.96 (0.33,2.74) | 0.66 |
| ER-/PR- | 320/39 | 1.16 (0.47,2.86) | 178/22 | 1.16 (0.32,4.18) | 142/17 | 2.17 (0.40,11.80) | 0.75 |

Hazard ratios (HR) and 95% confidence intervals (95% CI) from delayed-entry Cox proportional hazard models with 5-year follow-up. All models are adjusted for age at diagnosis, prognostic factors (tumor size, nodal status, histological grading), BMI, smoking status, alcohol consumption, Charlson Comorbidity Index, and stratified by study region and ER/PR-status. A 1-unit increase in the log_2_ transformed 27-HC concentration corresponds to a doubling. P_het_ comparing high estradiol subgroup vs. low estradiol subgroup. Abbreviations: BC=breast cancer; 27-HC=27-hydroxycholesterol; nM=nanomolar; ER=estrogen receptor; PR=progesterone receptor

### Table S3: Association between circulating 25-HC and breast cancer prognosis: 5-year survival

| 25-HC | | | | | | |  |
| --- | --- | --- | --- | --- | --- | --- | --- |
|  | **Overall** | | **Estradiol < 0.08 nM** | | **Estradiol ≥ 0.08 nM** | |  |
|  | **n/events** | **HR (95% CI),  per 1 unit increase** | **n/events** | **HR (95% CI),  per 1 unit increase** | **n/events** | **HR (95% CI),  per 1 unit increase** | **P_het_ (estradiol)** |
| All-cause mortality |  |  |  |  |  |  |  |
| Overall | 2265/139 | 0.89 (0.72,1.10) | 1132/62 | 1.07 (0.79,1.46) | 1133/77 | 0.78 (0.60,1.02) | 0.21 |
| ER+/PR+ | 1945/94 | 0.94 (0.74,1.19) | 954/35 | 1.02 (0.71,1.47) | 991/59 | 0.86 (0.62,1.18) | 0.44 |
| ER-/PR- | 320/45 | 0.79 (0.52,1.19) | 178/27 | 1.33 (0.66,2.66) | 142/18 | 0.44 (0.23,0.85) | 0.09 |
| BC-specific mortality |  |  |  |  |  |  |  |
| Overall | 2265/87 | 0.92 (0.71,1.20) | 1132/40 | 1.21 (0.84,1.75) | 1133/47 | 0.79 (0.57,1.09) | 0.11 |
| ER+/PR+ | 1945/50 | 1.01 (0.72,1.41) | 954/19 | 1.16 (0.73,1.82) | 991/31 | 0.89 (0.57,1.37) | 0.29 |
| ER-/PR- | 320/37 | 0.78 (0.50,1.22) | 178/21 | 1.53 (0.72,3.28) | 142/16 | 0.45 (0.23,0.92) | 0.08 |
| Recurrence |  |  |  |  |  |  |  |
| Overall | 2265/90 | 0.95 (0.73,1.23) | 1132/41 | 1.21 (0.84,1.75) | 1133/49 | 0.81 (0.59,1.13) | 0.13 |
| ER+/PR+ | 1945/51 | 1.03 (0.74,1.44) | 954/19 | 1.16 (0.73,1.82) | 991/32 | 0.92 (0.59,1.42) | 0.36 |
| ER-/PR- | 320/39 | 0.79 (0.51,1.23) | 178/22 | 1.53 (0.72,3.28) | 142/17 | 0.44 (0.23,0.86) | 0.09 |

Hazard ratios (HR) and 95% confidence intervals (95% CI) from delayed-entry Cox proportional hazard models with 5-year follow-up. All models are adjusted for age at diagnosis, prognostic factors (tumor size, nodal status, histological grading), BMI, smoking status, alcohol consumption, Charlson Comorbidity Index, and stratified by study region and ER/PR-status. A 1-unit increase in the log_2_ transformed 25-HC concentration corresponds to a doubling. P_het_ comparing high estradiol subgroup vs. low estradiol subgroup. Abbreviations: BC=breast cancer; 25-HC=25-hydroxycholesterol; nM=nanomolar; ER=estrogen receptor; PR=progesterone receptor
